# Comparison of effectiveness of hyaluronic acid and melatonin with guided tissue regeneration (GTR) membrane versus guided tissue regeneration (GTR) membrane alone in the treatment of intra-bony defects in human subjects: A split-mouth randomised controlled clinical trial

**DOI:** 10.1016/j.jobcr.2026.01.001

**Published:** 2026-01-16

**Authors:** Subasree Soundarajan, Sankari Malaiappan

**Affiliations:** Department of Periodontics, Saveetha Dental College and Hospitals, Saveetha Institute of Medical and Technical Sciences, Chennai, 600077, Tamil Nadu, India

**Keywords:** Chronic periodontitis, Drug, Green product, Intrabony defects, Hyaluronic acid, Melatonin

## Abstract

**Background:**

Melatonin is one substance that is primarily generated by the pineal gland and numerous other tissues and has vital involvement in many physiological processes, including bone remodeling. Over the last several years, another emerging molecule serving as a potential candidate for periodontal regeneration is the hyaluronic acid (HA), a key extracellular matrix component involved in cell migration. The aim of this study is the additive effect of hyaluronic acid and melatonin with GTR membrane in intrabony defects among patients with chronic periodontitis.

**Materials and methods:**

15 bilateral intra-bony defects were treated either with Hyaluronic acid and melatonin along with GTR membrane (Group I), or GTR membrane alone (Group II). Clinical and radiographic parameters assessed were pocket depth, clinical attachment level, and volume of bone defect at baseline and 6 months after the intervention. The biochemical parameter assessed was Matrix metalloproteinase - 1 (MMP-1) and Tissue inhibitor of metalloproteinase (TIMP-1) at baseline and one month after the intervention.

**Results:**

Both the study groups showed statistically significant differences in all the parameters from baseline to follow-up. However, group 1 (Hyaluronic acid + Melatonin + GTR membrane) showed statistically significant improvement in all the study parameters when compared to group 2 (GTR alone).

**Conclusion:**

It can be concluded that the combination of hyaluronic acid and melatonin was effective in the regeneration of intrabony defects among patients with chronic periodontitis. However, further studies with a longer time period and with additional microbiological assessment would be desirable so as to substantiate the current results.

## Teaser

Combination of hyaluronic acid and melatonin was effective in regeneration of intrabony defects among patients with chronic periodontitis in terms of probing depth, clinical attachment level, bone fill, MMP-1 and TIMP-1 levels.

## 1 Introduction

Periodontal disease, characterized by inflammation affecting the gingiva, alveolar bone, and periodontal ligament, is primarily initiated by a shift in microbial flora.^1^ The inflammatory response of the host plays a pivotal role in the disease's progression and subsequent tissue destruction. A notable event in periodontitis development is the conversion of junctional epithelium to pocket epithelium, influenced either directly by microorganisms or indirectly by the host tissue response.^2^ The interaction between microorganisms and the host determines the course and extent of the disease.^3^ Conventional mechanical debridement alone cannot eliminate all periodontopathic bacteria, particularly those in challenging areas like grooves, furcations, concavities, and deep pockets, presenting a clinical challenge, especially with pockets associated with deep intra-bony defects.^4^

Numerous regenerative techniques have been explored over the years to restore compromised teeth affected by periodontal disease.^5^ The goal of regenerative therapy is to rehabilitate the lost periodontal tissues. Various regenerative approaches involve bone grafts, guided tissue regeneration, platelet concentrates, and growth factors.^6^ Complete regeneration necessitates a combination of materials with anti-inflammatory and antioxidant properties, along with a scaffold serving as an extracellular matrix^7^^,^^8^.

Melatonin, an endogenous hormone synthesized primarily by the pineal gland, has regenerative potential and is involved in bone remodeling, dental implant osseointegration, and addressing osteoporosis.^9^ Studies have demonstrated melatonin's role in enhancing the proliferation of pre-osteoblasts, osteoblasts, and osteoblast-like cells, promoting the expression of type I collagen and bone marker proteins, and facilitating the formation of the mineralized matrix.^10^ Melatonin also influences energy metabolism and exhibits anti-inflammatory and antioxidant actions 11, 12, 13, 14, 15. Its anti-inflammatory effects are attributed to the regulation of pro- and anti-inflammatory cytokines in various pathophysiological conditions, contributing to bone formation by stimulating osteoblast proliferation and functioning as an osteoconductive scaffold.^16^

Both hyaluronic acid (HA) and melatonin are known for their favorable influence on periodontal wound healing. HA contributes to angiogenesis, fibroblast migration, and extracellular matrix stabilization, while melatonin exerts potent antioxidant, anti-inflammatory, and osteopromotive effects. The rationale behind combining these two biomolecules lies in their complementary biological mechanisms—HA's regenerative scaffold properties and melatonin's modulation of bone metabolism and oxidative stress. Their combined application with GTR may therefore yield an additive regenerative effect that enhances bone fill and clinical outcomes compared to GTR alone. Although both HA and melatonin have been individually studied, their combined use in humans remains limited in periodontal regeneration. The hypothesis tested was that the combination therapy would result in significantly greater clinical attachment gain, defect fill, and early biochemical modulation than GTR alone. Although the study did not include separate HA or melatonin control groups, it aimed to specifically evaluate their additive effect with GTR.

Tissue growth involves not only intracellular processes but also interactions between cells and the extracellular matrix 17, 18, 19. Hyaluronic acid (HA), a natural component of the extracellular matrix, is used in regenerative treatments due to its ability to mimic the extracellular environment.^20^^,^^21^ Biomimetic ECM incorporation enhances cell adhesion, proliferation, and differentiation. HA, known for its role in inflammation, granulation tissue formation, epithelial development, and tissue remodeling, is postulated to contribute to periodontal wound healing.^22^^,^^23^ Nonsurgical periodontal therapy, incorporating HA, demonstrates anti-inflammatory, anti-edematous, and antibacterial effects.^24^ The supplementary application of hyaluronic acid (HA) in non-surgical approaches to periodontal care, such as scaling and root planing (SRP), has been associated with improved clinical results, including reduction in bleeding and probing depth 25, 26, 27. Nevertheless, when HA is administered subgingivally as an adjunct to SRP in chronic periodontitis cases, there is a lack of significant changes in the bacterial composition of subgingival plaque sentence as per reviewer suggestion.^28^ Significantly, incorporating hyaluronic acid as a supplementary component in periodontal surgical procedures has demonstrated favorable results with respect to clinical parameters 29, 30, 31, 32, 33, 34, 35, 36, 37, 38.

The current study aims to assess the combined impact of hyaluronic acid and melatonin when used in conjunction with a GTR membrane for treating intrabony defects in individuals diagnosed with chronic periodontitis.

## 2 Materials and method

**Fig. 1:**
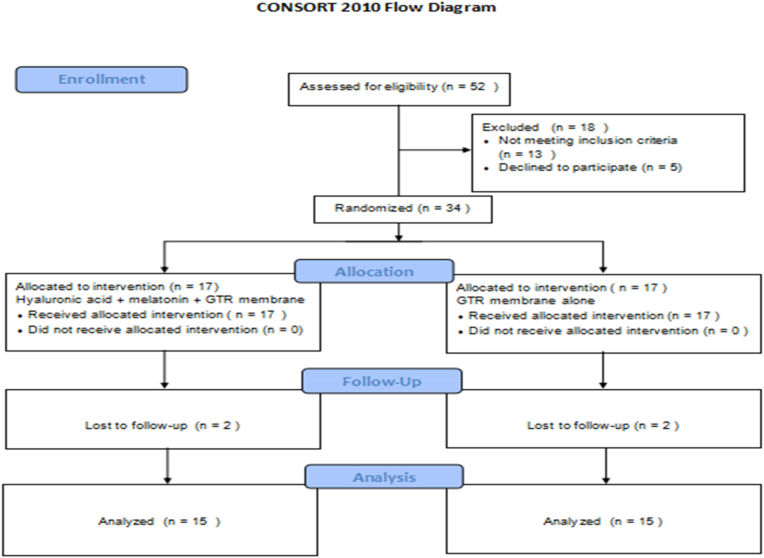
CONSORT flow chart.

A prospective, randomized controlled split-mouth clinical trial was designed to investigate the additional impact of hyaluronic acid and melatonin, along with a GTR membrane, on bone regeneration in intrabony periodontal defects. This study adhered to the principles of the Helsinki Declaration of 1975, as revised in 2013. Participants for the study were enlisted from individuals seeking outpatient care at the Department of Periodontics at Saveetha Dental College in Chennai, India, adhering to pre-established inclusion and exclusion criteria. A detailed explanation of the planned treatment was provided to all patients, and their informed consent was obtained. The study's design is depicted following the CONSORT flowchart, presented in Fig. 1.

## 3 Inclusion criteria

Individuals aged between 20 and 60 years, diagnosed with chronic periodontitis according to the 1999 American Academy of Periodontology classification, were eligible for inclusion. The criteria for inclusion involved the presence of one or two pairs of contralateral intrabony pockets associated with maxillary or mandibular molars, exhibiting a probing depth (PD) equal to or greater than 5 mm and clinical attachment loss (CAL) equal to or exceeding 4 mm. Additionally, three dimensional radiographic measurement of the two or three walled defects was done using cone-beam computed tomography.

## 4 Exclusion criteria

Individuals with pre-existing systemic illnesses, those taking medications known to influence the results of periodontal therapy or affect platelets, individuals on drugs that impede blood clotting, smokers, pregnant women, individuals with mobile teeth, excessive occlusal contacts, carious teeth, and loss of vitality in the affected area/tooth were considered exclusion criteria for this study.

## 5 Sample size calculation

Based on the study done by et al., sample calculation was done using G Power with power and α error at 90 and 0.05 respectively, which showed that a minimal sample size of 15 was required in each group.^39^

## 6 Randomization and allocation concealment

Lottery method was used for randomization. Lots were prepared in which the tooth number in relation to the intrabony pocket was mentioned. Both the lots representing each of the contralateral sites were placed in an envelope. A person other than the operator was asked to pick up one slip from the envelope, and the site which was picked up was allotted to group I (Hyaluronic acid + Melatonin + GTR membrane), and the other contra lateral site was allotted to group II (GTR membrane alone).

## 7 Armamentarium used

Mouth mirror, UNC 15 Pressure sensitive periodontal probe, Shepherd's hook explorer, Tweezer, Ultrasonic scaler, Kidney tray, Bard Parker handle, No. 15 Bard Parker blade, Periosteal elevator, Area specific Gracey curettes (Hu- Friedy), Tissue forceps, Glass dappen dish, 3-0 non-absorbable silk suture, needle holder and surgical scissors.

## 8 Clinical measurements

The probing depth (PD) and clinical attachment level (CAL) were measured with a pressure-sensitive University of North Carolina No 15 (UNC-15) probe. To standardize the positioning of the probe and ensure precise placement at various time points, custom-made acrylic stents with grooves were employed.

## 9 Radiographic measurements

In all instances, a CBCT scanner equipped with a flat panel detector (CS 9600 Carestream flat panel detector) was utilized, with an exposure volume configured at 102 mm in diameter and 102 mm in height. The manufacturer's recommended settings of 120 kV and 6.3 mA were applied, with an exposure duration of 15 s and a total exposure of 796 mGy/cm^2^. The assessment of defect volume was conducted using the medical image viewer in DICOM viewer through live-wire freehand modeling. Measurements of defect volume were taken at both baseline and 6 months.^40^

## 10 GCF sample collection

The levels of MMP-1 and TIMP-1 were assessed in gingival crevicular fluid (GCF) at two time points: baseline (before periodontal flap surgery) and 1 month postoperatively. Before GCF collection, supra gingival plaque was removed from each tooth using cotton pellets. Cotton rolls were employed to isolate and gently air dry individual tooth sites. GCF was collected by placing calibrated capillary tubes at the gingival crevice entrance. Subsequently, 1 μl of GCF was transferred into an Eppendorf tube containing 98 μl of Phosphate Buffered Saline (PBS) and 1 μl of a protease inhibitor cocktail using a jet of air pressure from the capillary tube. The samples were then stored at −20 °C.

## 11 Matrix metalloproteinase 1 & tissue inhibitor of metalloproteinase 1 levels analysis

Gingival crevicular fluid (GCF) concentrations of MMP-1 and TIMP-1 were determined using enzyme-linked immunosorbent assay (ELISA) with commercially available kits (RayBiosHuman MMP-1 ELISA Kit and RayBiosHuman TIMP-1 ELISA Kit) specifically designed for human MMP-1 and human TIMP-1. The GCF samples were suitably diluted (at a ratio of 1:40 for MMP-1 and 1:100 for TIMP-1) using the provided diluent in the ELISA kit. All procedures of the assay were carried out in accordance with the manufacturer's instructions.

After preparation, both samples and standards were incubated for 2.5 h at room temperature in wells pre-coated for this purpose. The solution was then discarded, and each well underwent four washes. A 100 μl aliquot of the prepared biotin antibody was added and incubated for 1 h at room temperature. Following another round of washing, 100 μl of the prepared HRP Streptavidin solution was added and left to incubate for 45 min at room temperature. After a final wash, 100 μl of tetramethylbenzidine one-step substrate reagent was added and allowed to incubate for 30 min at room temperature. The reaction was terminated by adding 50 μl of Stop Solution to each well, and the optical densities of both standards and samples were promptly measured at a wavelength of 450 nm.

## 12 Clinical procedures

### 12.1 Pre surgical procedure

This clinical investigation adopted a randomized controlled trial design with a split-mouth configuration. Site selection involved random allocation into two groups: Group I (Hyaluronic acid + Melatonin + GTR membrane) and Group II (GTR alone), employing a lottery method. Following an initial comprehensive examination and treatment planning, participants received meticulous instructions on oral hygiene practices geared towards plaque control. Scaling and root planing carried out was systematically. Participants were further directed to incorporate a 0.2 % chlorhexidine mouth rinse into their daily routine, twice daily, for a prescribed two-week period following SRP.

### 12.2 Hyaluronic acid and melatonin preparation

5 mg of melatonin powder was measured using a laboratory weight measuring instrument. It was then mixed with 0.8 % Hyaluronic acid gel (Gengigel ®). Melatonin used was pharmaceutical-grade, Sigma-Aldrich, USA, purity ≥98 %.

### 12.3 Surgical procedure

Group 1 sites underwent treatment involving Hyaluronic acid and melatonin in combination with a resorbable guided tissue regeneration membrane (Periocol-GTR) post open flap debridement (OFD). Conversely, Group 2 sites on the contralateral side received treatment solely with the GTR membrane following OFD. Preceding the operation, patients were instructed to perform preoperative 0.2 % chlorhexidine rinses.

**Fig. 2:**
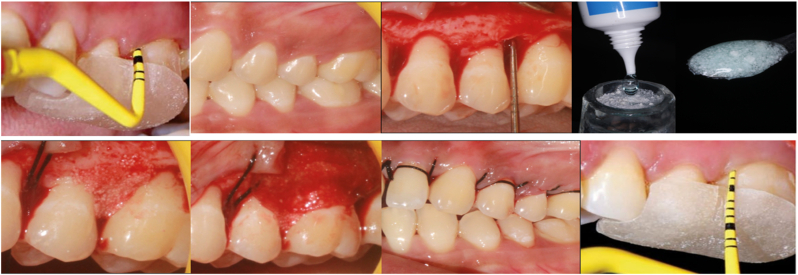
Surgical procedure.

Following the administration of local anesthesia (lignocaine with adrenaline 1:2,00,000), Kirkland flap was elevated. Using area specific curettes, root planing followed by debridement of the defect was carried out. The mixture of Hyaluronic acid gel and melatonin was packed into the defect and GTR membrane placed over it in Group I, GTR membrane alone was placed in Group II defects. Flap was secured after repositioning the mucoperiosteal flap using 3-0 non-absorbable silk suture. Following the procedure, patients were provided with postoperative prescriptions for non-steroidal anti-inflammatory medication and systemic antibiotics. Also, postoperative instructions were given. After 1 week, the sutures were removed and saline irrigation done. The surgical procedure is depicted in Fig. 2.

**Fig. 3:**
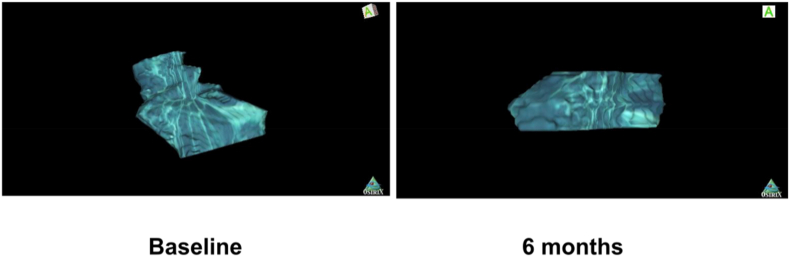
Improvement in defect volume in CBCT in the test group at 6 months.

### 12.4 POST-SURGICAL measurements

Six months post-surgery, a clinical and radiographic assessment was conducted (see Fig. 3). Probing depth and clinical attachment level measurements were recorded using acrylic stents that had been employed previously. Baseline and one-month post-surgery evaluations included the assessment of MMP-1 and TIMP-1 levels in gingival crevicular fluid (GCF).

## 13 Statistical analysis

The data obtained were recorded into a spreadsheet and subjected to comprehensive analysis using SPSS for Windows (Statistical Package for the Social Sciences, version 21) within the context of the research. Inter-group comparisons were evaluated through an independent *t*-test, while intra-group evaluations were performed using a paired *t*-test to scrutinize parameters across distinct time intervals within each group. A predetermined threshold of statistical significance was established at p < 0.05 for all conducted tests.

## 14 Results

Within the framework of this randomized controlled clinical trial, 17 bilateral intra-bony defects were subject to treatment using either Hyaluronic acid + melatonin + GTR membrane (Group I, n = 17) or GTR membrane alone (Group II, n = 17). Due to two dropouts, 15 bilateral intra-bony defect sites were diligently followed throughout the study duration. No discernible adverse effects were clinically evident following the intervention among the enrolled patients.

The mean and standard deviation of age, as well as the percentage distribution of male and female participants, were calculated. The average age of patients was 38.8 ± 6.2 years, with 62 % being male per reviewer comment and 38 % female.

Clinical parameters such as probing depth and clinical attachment level, along with radiographic parameters such as defect volume in CBCT, were assessed at baseline and 6 months. Biochemical parameters, specifically MMP-1 and TIMP-1 levels in GCF, were measured at baseline and one month after the intervention.

Normality of the data was confirmed through Shapiro-Wilk and Kolmogrov Smirnov tests, indicating normally distributed data. Consequently, parametric tests were selected. Independent t-tests were conducted for inter-group comparisons between Group I and Group II. Paired t-tests were employed for intra-group comparisons within Group I and Group II separately, evaluating changes from baseline to 6 months after intervention for PD, CAL, and defect volume, and from baseline to 1 month after intervention for MMP and TIMP-1 levels.

Intergroup comparison at baseline revealed no statistically significant differences, indicating a homogeneous study population (Table 1). Intergroup comparisons at 6 months after intervention and 1 month after intervention for various parameters demonstrated statistically significant differences between the two groups (Table 2).

**Table 1:** Inter-group comparison of PD, CAL, MMP-1, TIMP-1 and defect volume between different groups at baseline (Independent *t*-test).

VARIABLES	GROUP I (HYALURONIC ACID + MELATONIN + GTR MEMBRANE IN INTRA-BONY DEFECTS	GROUP II (GTR ALONE) IN INTRA-BONY DEFECTS	P VALUE
Probing depth	7.60 ± 1.59	7.26 ± 1.33	0.54
CAL	8.60 ± 1.63	8.20 ± 1.01	0.42
MMP-1 levels in GCF	1.77 ± 0.09	1.76 ± 0.09	0.71
TIMP-1 levels in GCF	26.50 ± 0.37	26.95 ± 0.37	0.98
Ratio of MMP-1 to TIMP-1	0.06 ± 0.004	0.06 ± 0.004	0.24
Defect volume	3.61 ± 0.14	3.65 ± 0.08	0.29

**Table 2:** Inter-group comparison of PD, CAL, MMP-1, TIMP-1 and defect volume between different groups at follow up (Independent *t*-test).

VARIABLES	GROUP I (HYALURONIC ACID + MELATONIN + GTR MEMBRANE IN INTRA-BONY DEFECTS	GROUP II (GTR ALONE) IN INTRA-BONY DEFECTS	P VALUE
Probing depth (6 months)	3.86 ± 1.30	5.93 ± 1.09	**0.04∗**
CAL (6 months)	5.20 ± 0.77	6.86 ± 0.74	**0.02∗**
MMP-1 levels in GCF (1 month)	1.10 ± 0.08	1.66 ± 0.09	**0.04∗**
TIMP-1 levels in GCF (1 month)	44.63 ± 1.50	35.60 ± 2.07	**0.04∗**
Ratio of MMP-1 to TIMP-1 (1 month)	0.02 ± 0.00	0.04 ± 0.00	**0.03∗**
Defect volume	2.08 ± 0.04	2.78 ± 0.11	**0.03∗**

Intra-group comparisons for Group I exhibited a statistically significant reduction in PD, CAL, and defect volume at 6 months after intervention and a significant reduction in MMP-1 and an increase in TIMP-1 at 1 month after intervention (p < 0.05) (Table 3) (see Table 4).

**Table 3:** Intra-group comparison within Group I (Hyaluronic acid + Melatonin) between baseline and follow-up (Paired *t*-test).

VARIABLES	Time points	Mean ± SD	P VALUE
Probing depth	Baseline	7.60 ± 1.59	**0.000∗**
	At 6months	3.86 ± 1.30	
CAL	Baseline	8.60 ± 1.63	**0.000∗**
	At 6months	5.20 ± 0.77	
MMP-1 levels in GCF	Baseline	1.77 ± 0.09	**0.000∗**
	At 1month	1.10 ± 0.08	
TIMP-1 levels in GCF	Baseline	26.50 ± 0.37	**0.000∗**
	At 1 month	44.63 ± 1.50	
Ratio of MMP-1 to TIMP-1	Baseline	0.06 ± 0.004	**0.000∗**
	At 1 month	0.02 ± 0.00	
Defect volume	Baseline	3.61 ± 0.14	**0.000∗**
	At 6months	2.08 ± 0.04

**Table 4:** Intra-group comparison within Group II (GTR alone) between baseline and follow-up (Paired *t*-test).

VARIABLES	Time points	Mean ± SD	P VALUE
Probing depth	Baseline	7.26 ± 1.33	**0.02∗**
	At 6months	5.93 ± 1.09	
CAL	Baseline	8.20 ± 1.01	**0.02∗**
	At 6months	6.86 ± 0.74	
MMP-1 levels in GCF	Baseline	1.76 ± 0.09	**0.02∗**
	At 1month	1.66 ± 0.09	
TIMP-1 levels in GCF	Baseline	26.95 ± 0.37	**0.02∗**
	At 1 month	35.60 ± 2.07	
Ratio of MMP-1 to TIMP-1	Baseline	0.06 ± 0.004	**0.02∗**
	At 1 month	0.04 ± 0.00	
Defect volume	Baseline	3.65 ± 0.08	**0. 02∗**
	At 6months	2.78 ± 0.11	

Similarly, intragroup comparisons for Group II revealed a statistically significant reduction in PD and CAL at 6 months after intervention, as well as a significant reduction in MMP-1 and an increase in TIMP-1 at 1 month after intervention. A statistically significant reduction in defect.

Cone-beam computed tomography (CBCT) evaluation demonstrated a marked reduction in defect volume in the test group (GTR + HA + melatonin) between baseline and 6 months. The reconstructed three-dimensional CBCT image (Fig. 2) clearly depicts bone fill and volumetric reduction of the intrabony defect, indicating favorable regenerative response. Representative CBCT sections of the test site are shown below, illustrating the volumetric change from baseline to 6 months.

## 15 Discussion

Periodontitis is a persistent inflammatory condition that leads to the deterioration of the supporting structures within the periodontium. It is primarily mediated by biofilm and the host response, which eventually leads to alveolar bone loss and intrabony defects.

The radiographic findings in this study were substantiated through CBCT-based volumetric evaluation, which provided three-dimensional confirmation of bone fill and defect reduction. The enhanced bone regeneration observed in the combination group aligns with the biological potential of HA and melatonin to promote osteoblastic differentiation, angiogenesis, and antioxidant protection at the healing site. The use of CBCT represents a significant methodological strength, offering superior accuracy compared to conventional intraoral periapical radiographs used in previous studies. The three-dimensional reconstruction (Fig. 2) provided visual and quantitative confirmation of defect fill, complementing the clinical and biochemical improvements observed in the test group. The ability of CBCT to capture volumetric bone changes with high spatial resolution represents a methodological strength of this study, offering more accurate assessment than conventional periapical radiographs. Although only representative CBCT images of the test group are presented, the contralateral control sites exhibited comparatively lesser bone fill on linear CBCT measurements, in line with the clinical outcomes.

The present study demonstrates several important methodological and clinical strengths that enhance the reliability and relevance of the findings. The split-mouth randomized controlled design is a key methodological advantage, markedly reducing inter-patient variability and ensuring that both treatment modalities were assessed under comparable biological and anatomical conditions. The use of standardized acrylic stents, pressure-sensitive probes, and calibrated measurements further improved consistency and minimized examiner-related errors. Additionally, the incorporation of CBCT-based three-dimensional volumetric analysis represents a significant clinical and methodological strength, offering superior accuracy and spatial resolution compared to traditional two-dimensional radiographs. This advanced imaging modality enabled precise quantification of defect morphology and bone fill, providing a more robust assessment of regenerative outcomes. Clinically, the novel combination of hyaluronic acid and melatonin with a GTR membrane reflects a biologically driven, minimally invasive regenerative approach that integrates scaffold stability, antioxidant activity, and osteopromotive effects. The inclusion of biochemical markers (MMP-1 and TIMP-1) adds further depth by capturing early host-response modulation, complementing the clinical and radiographic outcomes. Collectively, these methodological and clinical strengths enhance the credibility of the study and support the observed superiority of the combination therapy over GTR alone.

The effective restoration of periodontal tissues post-periodontal disease poses an ongoing challenge in medical research, despite considerable exploration and a nuanced understanding of periodontal tissue biology. Two primary strategies employed for periodontal regeneration are guided tissue regeneration (GTR) and tissue engineering approaches.^41^ GTR, a well-established clinical method for periodontium regeneration, hinges on preventing epithelial apical growth over exposed root surfaces using a barrier membrane. This process fosters the development of periodontal ligament (PDL) tissues and alveolar bone by facilitating the proliferation of PDL cells and osteoblasts.^42^ Factors such as diabetes, smoking, dental plaque control, and tooth anatomy can significantly impact the outcomes of periodontal regeneration in medical research.

Periodontal researchers are actively exploring innovative regenerative materials, with a particular focus on combination therapy. This strategy involves the simultaneous application of various reconstructive treatments for periodontal issues to achieve additive per reviewer: replaced ‘synergistic’ with ‘additive’ effects. Typically, combinations of bone grafts, guided tissue regeneration membranes, and growth enhancement factors like Platelet Rich Fibrin (PRF) and Concentrated Growth Factor (CGF) are widely endorsed. However, the combination of melatonin particles as a bone graft and hyaluronic acid as the carrier has not been employed in periodontal regenerative procedures. Combination therapy is generally considered highly reliable and successful, especially in managing intrabony defects, as it integrates different regenerative principles, including conductivity and inductivity, space provision, wound stability, matrix development, and cell differentiation.

This study specifically investigates the regenerative potential of hyaluronic acid when combined with melatonin for managing intrabony defects. Recent histological animal investigations on hyaluronic acid for periodontal regeneration have shown promising results, indicating its potential to stimulate periodontal regeneration in different defect types 43, 44, 45. This effect is thought to be mediated by interactions with CD44 during the early stages of periodontal tissue regeneration.

Melatonin, a hormone with accepted osteogenic effects through various mechanisms, serves as an additive material in this combination. While most studies on melatonin have been conducted in vitro and on animals, there is a scarcity of research on its effects in humans.^46^ This study aims to contribute valuable insights into the regenerative potential of combining hyaluronic acid and melatonin for the management of intrabony defects in the context of periodontal regeneration.^47^

Conducted as a prospective randomized controlled split-mouth clinical trial, the research involved 17 patients and 34 sites. Notably, two patients, each contributing four operative sites (two in the experimental group and two in the control group), withdrew from the study at the 6-month mark. Despite their inability to attend the 6-month follow-up due to personal reasons, these participants expressed satisfaction with the outcomes of the periodontal surgery. Consequently, the analysis focused on data from 30 sites (15 patients) that were successfully followed up until the completion of the study. The randomization process utilized online software (RandomAlloc.exe.Version 1.0), incorporating parameters such as the number of groups, patients per group, and numerical range to generate random numbers. The selected sites comprised intrabony defects associated with maxillary or mandibular first molars. Each site received a numerical assignment, dictating its allocation to either Group I (Hyaluronic acid + melatonin + PerioCol®-GTR membrane) or Group II (PerioCol®-GTR membrane alone).

The combination of hyaluronic acid and melatonin exhibited compatibility without any adverse effects in the subjects of this study. To measure the regenerative efficacy of melatonin, clinical parameters including pocket depth (PD), clinical attachment level (CAL), and radiographic parameters (defect volume assessment using CBCT) were examined at baseline and six months post-intervention. Furthermore, biochemical parameters, specifically MMP-1 and TIMP-1 levels in gingival crevicular fluid (GCF), were scrutinized using colorimetrically optimized kinetic test kits at baseline and one month.

Importantly, at baseline, there was no statistically significant difference observed in all the study parameters (PD, CAL, radiographic defect volume, MMP-1, and TIMP-1 levels) between the groups, indicating a homogeneous study population at the outset. However, at the follow-up, a statistically significant difference emerged in clinical parameters such as probing depth (PD), clinical attachment level (CAL), and bone defect volume, as well as MMP-1 and TIMP-1 levels between groups 1 and 2.

At the 6-month assessment, Group 1 (Hyaluronic acid + melatonin + PerioCol®-GTR membrane) exhibited a mean pocket depth (PD) of 3.86 ± 1.30, while Group 2 (PerioCol®-GTR membrane alone) showed a PD of 5.93 ± 1.09. Additionally, the mean clinical attachment level (CAL) at 6 months was 5.20 ± 0.77 for Group 1 and 6.86 ± 0.74 for Group 2. These results are consistent with a study where the treatment of intrabony defects with a combination of hyaluronic acid and a bone grafting material demonstrated superior clinical outcomes compared to hyaluronic acid alone. One possible explanation for these differences is that hyaluronic acid (HA), similar to other biologics, has a fluid consistency that may impede its ability to create sufficient space for effective periodontal regeneration.^48^^,^^49^ This characteristic could lead to the collapse of the mucoperiosteal flap, limiting the success of regenerative surgery. Combining HA with a bone grafting material may address this issue by providing robust support to the mucoperiosteal flap, preventing its collapse into the defect, thereby ensuring clot stability and offering the necessary space for periodontal regeneration.^50^

In the current investigation, Group 1 exhibited a statistically significant decrease in pocket depth (PD) compared to Group 2. These results are in line with prior studies examining the effectiveness of incorporating melatonin with scaling and root planing (SRP) in managing intrabony defects.^51^^,^^52^ This congruence in findings could be attributed to melatonin's anti-inflammatory properties, which are associated with its role as a scavenger for both exogenous and endogenous reactive oxygen species (ROS) and reactive nitrogen species.^53^

The primary cause of periodontal destruction is likely attributed to matrix metalloproteinases (MMPs) derived from host cells.^54^ MMP-1, in particular, is suggested to play a crucial role in initiating collagen degradation in periodontal disease.^55^^,^^56^ Previous studies have reported an increased expression of MMP-1 mRNA in inflamed gingival tissue of individuals with chronic periodontitis compared to healthy controls^57^, ^58^, ^59^, ^60^. Tissue inhibitors of metalloproteinases (TIMPs) bind to MMPs, irreversibly inhibiting their enzyme activity. Altered TIMP expression is a known occurrence in various disease processes, influencing the processing of the extracellular matrix.^61^ There is speculation that TIMP-1 may be more effective in inhibiting MMP-1.^62^ Thus, this study aimed to investigate changes in gingival crevicular fluid (GCF) levels of MMP-1 and TIMP-1 in individuals with both periodontal health and disease. ELISA was selected as the method for detecting GCF levels of MMP-1 and TIMP-1 in the present study.

Results obtained in the current study revealed a statistically significant reduction in the level of matrix metalloproteinase-1 (MMP-1) in gingival crevicular fluid (GCF) and a concurrent increase in tissue inhibitor of metalloproteinase-1 (TIMP-1) levels from baseline to one month postoperatively in both experimental groups. Furthermore, Group 1 exhibited a notably higher reduction in MMP-1 levels (1.10 ± 0.08) compared to Group 2 (1.66 ± 0.09), and a more substantial increase in TIMP-1 levels (44.63 ± 1.50) compared to Group 2 (35.60 ± 2.07). The MMP-1/TIMP-1 ratio was significantly elevated in subjects from Group 2 (0.04) compared to Group 1 (0.02). These findings suggest that maintaining a balanced ratio of GCF MMP-1 and TIMP-1 levels plays a crucial role in preserving a healthy periodontal condition. This observation is consistent with previous studies that assessed MMP-1 and TIMP-1 levels before and after nonsurgical periodontal therapy in individuals with chronic periodontitis 63, 64, 65, 66. While the observed reduction in MMP-1 levels and improvement in the MMP-1/TIMP-1 balance at 1 month suggest favorable modulation of the early healing environment, these findings should be interpreted within the context of short-term biochemical behavior. Because biomarkers were assessed only once, the study cannot establish a direct causal link between early molecular changes and the 6-month clinical improvements. Thus, the biochemical results support—but cannot definitively explain—the clinical outcomes. Additional studies with serial biomarker measurements are required to confirm the trajectory of inflammatory and remodeling responses associated with laser-assisted regenerative therapy.

In this study, a substantial and statistically significant reduction in defect volume was observed in Group 1 (Hyaluronic acid + melatonin + PerioCol®-GTR membrane - 2.08 ± 0.04) compared to Group 2 (PerioCol®-GTR membrane - 2.78 ± 0.11) at the 6-month follow-up, with a p-value below 0.05 threshold per reviewer comment. This finding aligns with a separate study where Group II (1 % melatonin gel - 1.46 ± 0.58 mm) exhibited a greater amount of bone fill compared to Group I (Placebo - 0.50 ± 0.38 mm) at the 6-month mark (P < 0.0001).^67^ Consistent results were also noted in two other animal studies,^68^^,^^69^ where in entire periodontal bone defect was filled with new lamellar bone and a narrow marrow.^70^^,^^71^

To our knowledge, this is the first study to evaluate the cumulative impact of melatonin combined with hyaluronic acid using a guided tissue regeneration (GTR) membrane in intrabony defects, in comparison to GTR membrane alone. Notably, radiographic evaluation was conducted employing cone-beam computed tomography (CBCT), marking a departure from previous studies that utilized intraoral periapical radiographs.^72^

The utilization of locally administered melatonin in conjunction with hyaluronic acid exhibited a substantial enhancement across all clinical and radiographic parameters in our current investigation. This combination emerges as an innovative integration into conventional periodontal treatment strategies, offering improved outcomes supported by well-established immunomodulatory, anti-inflammatory, and potent antioxidant attributes. Furthermore, its application in periodontal bony defects shows promise for aiding bone healing and facilitating new bone formation. Additionally, considering the acknowledged efficacy of hyaluronic acid as a soft tissue filler, it presents an additional advantage in potentially averting the loss of interdental papilla following flap surgery. Acknowledging the novelty of this combination in the realm of regenerative biomaterials, it's crucial to recognize that multiple factors may have contributed to the observed outcomes. Despite the strengths of the study, certain limitations must be acknowledged. Biomarker evaluation was performed only at the 1-month postoperative interval, which provides insight primarily into early healing events. The absence of additional early (e.g., 1–2 weeks) and late (3–6 months) time points limits the ability to comprehensively map the temporal dynamics of MMP-1 and TIMP-1 expression and to correlate these biochemical findings with long-term clinical outcomes. Therefore, the 1-month biomarker results should be interpreted cautiously and not as definitive evidence of long-term periodontal regeneration. Future studies incorporating multiple time-point analyses and larger sample sizes may provide deeper understanding of the host response in regenerative therapy.

## 16 Conclusion

Within the limitations of this study, it can be concluded that the combination of hyaluronic acid and melatonin with guided tissue regeneration resulted in superior clinical and radiographic outcomes compared to GTR alone. The use of CBCT further confirmed the enhanced bone fill, suggesting that this combination therapy may serve as a promising adjunct in periodontal regenerative therapy.

## Author's contribution

All authors have made substantial contributions to conception and design of the study. Subasree Soundarajan have been involved in data collection and data analysis. Subasree Soundarajan and Sankari Malaiappan have been involved in data interpretation, drafting the manuscript and revising it critically and have given final approval of the version to be published.

## Ethical approval

Approval for this study was obtained from the human subjects ethics board of Saveetha Dental College (Approval number SRB/SDC/PERIO-1902/20/TH-01).

## Sources of funding

Self funded project.

## Declaration of competing interest

The authors declare the following financial interests/personal relationships which may be considered as potential competing interests: Subasree Soundarajan reports equipment, drugs, or supplies was provided by SIMATS Deemed University Saveetha Dental College. Subasree Soundarajan reports a relationship with SIMATS Deemed University Saveetha Dental College that includes: employment. Subasree Soundarajan has patent pending to Subasree Soundarajan. no conflict of interest If there are other authors, they declare that they have no known competing financial interests or personal relationships that could have appeared to influence the work reported in this paper.
